# Toward 3D Reconstruction of Outdoor Scenes Using an MMW Radar and a Monocular Vision Sensor

**DOI:** 10.3390/s151025937

**Published:** 2015-10-14

**Authors:** Ghina El Natour, Omar Ait-Aider, Raphael Rouveure, François Berry, Patrice Faure

**Affiliations:** 1 Lasmea-UMR UBP-CNRS 6602, Université Blaise Pascal, Aubière 63170, France; E-Mails: omar.ait-aider@univ-bpclermont.fr (O.A.-A.); berry@univ-bpclermont.fr (F.B.); 2 IRSTEA, Institut National de Recherche en Sciences et Technologies pour l’Environnement et l’Agriculture, Aubière 63170, France; E-Mails: raphael.rouveure@irstea.fr (R.R.); patrice.faure@irstea.fr (P.F.)

**Keywords:** 3D reconstruction, multi-sensor calibration, radar, vision

## Abstract

In this paper, we introduce a geometric method for 3D reconstruction of the exterior environment using a panoramic microwave radar and a camera. We rely on the complementarity of these two sensors considering the robustness to the environmental conditions and depth detection ability of the radar, on the one hand, and the high spatial resolution of a vision sensor, on the other. Firstly, geometric modeling of each sensor and of the entire system is presented. Secondly, we address the global calibration problem, which consists of finding the exact transformation between the sensors’ coordinate systems. Two implementation methods are proposed and compared, based on the optimization of a non-linear criterion obtained from a set of radar-to-image target correspondences. Unlike existing methods, no special configuration of the 3D points is required for calibration. This makes the methods flexible and easy to use by a non-expert operator. Finally, we present a very simple, yet robust 3D reconstruction method based on the sensors’ geometry. This method enables one to reconstruct observed features in 3D using one acquisition (static sensor), which is not always met in the state of the art for outdoor scene reconstruction. The proposed methods have been validated with synthetic and real data.

## 1. Introduction

Outdoor 3D reconstruction is a challenging aspect in many applications, such as mapping, autonomous navigation and localization, disaster control and many others. The evolution in computer science technologies, the decreasing sensor prices and the increasing number of applications referring to the 3D representation of the environmenthas pushed forward research in the field of 3d cartography of large outdoor environments. Methods existing in the literature are based on vision or range sensors or a combination of these two sensors. In this regard, a combination of sensors is an obvious solution to overcome the limitations of single sensors. Thereby, multi-sensory fusion has been recently a point of interest in widespread applications and research, especially for 3D mapping applications [1,2,3].

Despite the large number of studies (a survey of 3D reconstruction is presented in [4,5]), there are still many challenges for fully automatic and real-time modeling processes together with high quality results, requiring more contributions. Acquiring, storing and matching processes are costly, both in terms of memory and time. Furthermore, Outdoor 3D reconstruction is a challenging aspect because of many limitations due to large-scale and unshaped features and bad illumination conditions. For these reasons, the proposal of a simple, robust and fast algorithm dedicated to complete such an objective represents a major interest for several applications. The authors in [6] provide a comprehensive overview of urban reconstruction.

Regarding the low cost and high spatial resolution of vision sensors, a huge number of vision-based approaches for 3D reconstruction has been proposed. Methods for 3D scene reconstruction from an image sequence can be grouped into two classes: structure-from-motion and dense stereo.

SfM delivers a set of registered cameras (or poses), as well as sparse 3D point clouds (scene structure). The 3D point clouds are obtained from sparse selected feature points, which are detected in each image and then matched across the image sequence. SfM provides sparse, but accurate poses and structure. In the second step, dense matching is used for surface reconstruction. The aim is to reconstruct scene surfaces by recovering the 3D information from all pixels in contrast to sparse methods. The geometrical information obtained in the previous step is used to make the dense matching both robust and efficient. Some examples can be found in [7,8,9,10,11,12,13]. In the last few years, many works intended to fill the gap between the two approaches in order to propose methods that may handle very large-scale outdoor scenes [14,15]. In [16], the authors presented a new method, which is called the “mask automatic detecting method”, using a camera driving recorder, and which provided better results compared to a typical SfM method. The results seems to be of good quality, though it recommends a large amount of input data and heavy algorithms, which make it not quite suitable for real-time processing. It is also known that camera-based methods for large scene reconstruction generally suffer from scale factor drift and loop closure problems. Beside, vision sensors present common drawbacks due to the influence of image quality and adverse illumination and weather conditions. For this reason, tapping into active sensors has become essential.

The capability of range sensors to work in difficult atmospheric conditions and the decreasing cost make them well suited for extended outdoor robotic applications. For example, the authors in [17] used a radio detection and ranging (RADAR) sensor for simultaneous localization and mapping (R-SLAM algorithm) applications in agriculture. However, the radar failed to recognize the elevation, shape, texture and size of the target. Two acquisitions from two different points of view of the same scene are often needed in order to achieve the 3D reconstruction. Many solutions based on the combination of depth and vision sensors are described in the literature. SLAM applications with Kinect are also numerous [18,19]. Yet, the performances for outdoor applications are generally limited due to the small depth range and sensitivity to the outdoor natural light. An example of this fusion can also be found in [20]: an automatic classification of raw data from light detection and ranging (LiDAR) in external environments and a reconstruction of 3D models of buildings are presented. LiDAR is widely used for urban scene reconstruction; for instance, in [21], the authors combined data from a 3D LiDAR and images to create geometric 3D models of the world. The authors in [22], also, recently, combined six cameras and one 2D laser for urban 3D reconstruction. LiDAR provides a large number of accurate 3D points from a narrow field of view. Other examples could be found in [23,24]. However, the alignment of the large amount of data requires heavy processing algorithms that can be memory and time consuming. Reconstructed scenes using point cloud-based methods generally have an unstructured representation and cannot be directly represented as connected surfaces. In our method, in contrast, matches of large dimensions of surfaces (patches in the image with the target or a set of targets in the radar image) will be done, as shown, for example, in Figure 22b,d. In addition, LiDAR is generally more expensive than radar. Furthermore, one of LiDAR’s weaknesses *versus* radar is that the data acquired by LiDAR are somehow affected by the external illumination and weather conditions (like water and dust particles and also extreme sunlight). A review of the use of mobile LiDAR in several applications and of the advantages and challenges of LiDAR for city reconstruction have been summarized in [25]. Even though radar is much less used in the literature, in particular for the 3D reconstruction, it presents several advantages for outdoor applications: radar is highly independent of the illumination and weather conditions, and several targets can be detected in the same beam thanks to the physical property of the transmitted wave. These advantages make the exploitation of radar in our system of sensors very interesting and were our first motivation to explore the combination of a panoramic millimeter wave (MMW) radar and a camera, in order to achieve a sparse 3D reconstruction of large-scale outdoor environments. Recently, this combination has been the subject of many studies so far reported in the literature, for on-road obstacle detection and vehicle tracking: in [26], a camera and radar were integrated with an inertial sensor to perform road obstacle detection and classification.

Other works on radar-vision fusion for obstacle detection can be found in the literature [27,28,29,30]. However, we are not aware of any work using radar and a camera only, for outdoor 3D reconstruction. More than data fusion, our main objective is to build a 3D sensor that can provide textured elevation maps. Therefore, a geometrical model of the sensors and a calibration technique should be provided.

The challenge is to take full advantage of the context of data fusion, appropriately exploiting the complementarity of optical and radar sensors: we rely on the fact that the distance of an object in 3D space to the system is given by the radar measurements having a constant range error with increasing distance: with frequency modulated continuous wave (FMCW) radar, the radar to target distance is obtained with the measurement of the frequency difference between the transmitted signal and the received signal. This beat frequency is small for short distances and larger for longer distances. The distance resolution is equivalent to a frequency resolution: this frequency resolution is independent of the distance and depends on the frequency measurement performance of the data acquisition and signal processing system. For this reason, the radar uncertainty zone is constant with respect to an increasing depth.

In multi-sensor systems, each sensor performs measurements in its own coordinate system. Thus, one needs to transform these measurements into a global coordinate system. Generally, a calibration step enables one to compute this transformation in order to make the reconstruction simpler. In related works, this calibration method is not explicitly described. Sugimoto *et al.* [31] used the reflection intensity from MMW radar and image sequences to achieve a radar-vision spatial calibration. This method is hard to implement, because all of the points should be positioned exactly on the radar plane, as explained in Section 4.1. Our goal is to simplify this tricky and important step, which is crucial for the matching process and the reconstruction accuracy. Therefore, first, we propose a technique that uses only a set of radar-to-image point matches. These points are positioned in front of the camera/radar system, and the distances between them are measured. Then, the acquisitions from the two sensors are done simultaneously, with overlapping fields of view. Then, in order to relax this constraint, the scene is captured by the sensors from multiple points of view. A non-linear geometrical constraint is derived from each match, and a cost function is built. Finally, the transformation between the radar and the camera frames is recovered by a Levenberg–Marquardt (LM)-based optimization.

Once the calibration parameters are computed, 3D reconstruction of any radar-vision matched target can be achieved. Indeed, the intersection point of a sphere centered on the radar frame origin and a light ray passing through the camera optical center is the 3D position of the object. This geometrical constraint provides a quadratic equation, with two possible solutions. The correct one is chosen to solve the 3D reconstruction problem. Therefore, a small amount of input data (single image and panoramic frame) is sufficient to achieve a sparse 3D map allowing, thereby, real-time processing. Therefore, our method is flexible, and it is operational in stationary and slow motion mode. This work is an extension of the work presented in [32]. The paper is organized as follows: In Section 2, we describe the camera and radar geometrical models. The 3D reconstruction problem is addressed in Section 3. Section 4 focuses on the calibration method. Finally, experimental results obtained with both synthetic and real data are presented and discussed in Section 5.

## 2. System Model

**Figure 1:**
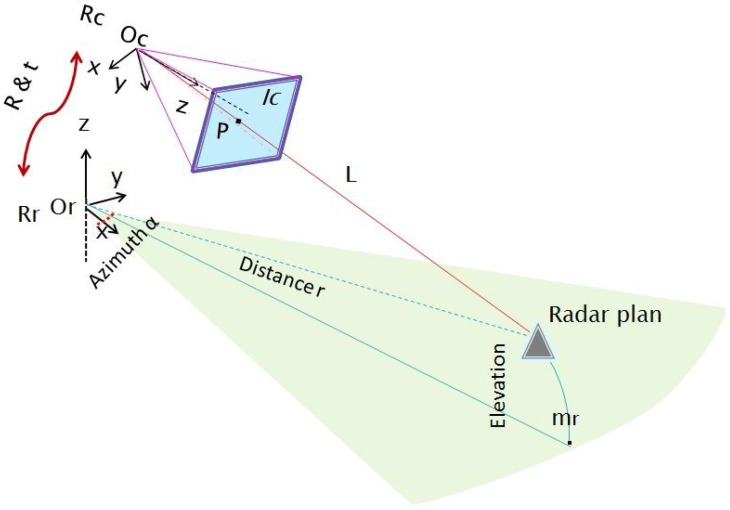
Sensors system geometry: $R_{c}$ and $R_{r}$ are the camera and radar frames, respectively. Polar coordinates $m_{r}\left(\alpha ,r\right)$ of the target are provided by the radar data, but not the elevation angle. The light ray *L* and the projected point *p* in the image $I_{c}$ are shown together with the horizontal radar plane.

The system model is formed by a camera and a radar that are rigidly linked. A standard pinhole model is assumed for the camera. The camera frame and center are denoted $R_{c}$ and $O_{c}\left(x_{O_{c}},y_{O_{c}},z_{O_{c}}\right)$, respectively. Similarly $R_{r}$ and $O_{r}\left(x_{O_{r}},y_{O_{r}},z_{O_{r}}\right)$ are respectively the radar’s frame and center. The sensors system is illustrated in Figure 1.

The radar performs acquisitions over $360^{\circ }$ per second thanks to its $1^{\circ }$ step rotating antenna. It generates a panoramic image each second, where detected targets are localized in 2D polar coordinates. The radar locates a target in range and angle. The radar to target distance measurement is based on the FMCW principle [33]. It can be shown that the frequency difference (called the beat frequency) between the transmitted signal and the signal received from a target is proportional to the sought distance. The reflected signal has a different frequency because of the continuous variation of the transmitted signal around a fixed reference frequency. The lateral resolution of the radar is about $4^{\circ }$ (see Table 1). The radar suffers from a low vertical resolution because of the wide angular aperture of the beam ($25^{\circ }$). The received signal echoes within this angular aperture (4× $25^{\circ }$), which is then integrated to form a single target.

Therefore, a 3D point detected by the radar is projected onto the horizontal plane passing through the center of the antenna first lobe of the radar, following a circular projection. Therefore, the real depth and azimuth of a detected target are provided without any altitude information. The projected point is denoted $m_{r}\left(\alpha ,r\right)$, where *α* and *r* are the polar coordinates of the point in the 3D space.

The camera performs a perspective projection relative to its optical center, consisting of two transformations: the first transformation projects a 3D point $\tilde{M}{\left(x,y,z,1\right)}^{T}$ into $\tilde{p}{\left(u,v,1\right)}^{T}$ (in a homogeneous coordinates system) in the image plane Ic, and it is written as follows:

(1) $$w\tilde{p}=\left[K|0\right]I_{4x4}\tilde{M}$$

(2) $$w\left[\begin{array}{c} u\\ v\\ 1 \end{array}\right]=\left[\begin{array}{cccc} -k_{u}f_{x} & s & u_{0} & |0\\ 0 & k_{v}f_{y} & v_{0} & |0\\ 0 & 0 & 1 & |0 \end{array}\right]\left[\begin{array}{cccc} 1 & 0 & 0 & 0\\ 0 & 1 & 0 & 0\\ 0 & 0 & 1 & 0\\ 0 & 0 & 0 & 1 \end{array}\right]\left[\begin{array}{c} x\\ y\\ z\\ 1 \end{array}\right]$$

where *w* is a scale factor representing the depth of $\tilde{M}{\left(x,y,z,1\right)}^{T}$ relative to the camera. *K* is the matrix of intrinsic parameters, assumed to be known, since the camera is calibrated using the MATLAB toolbox of [34]. The vertical and horizontal dimensions of a pixel of the optical photosensitive sensor are denoted $d_{x}$ and $d_{y}$, so $f_{x}=f/d_{x}$ and $f_{y}=f/d_{y}$. The principal point $pc(u_{0},v_{0})$ is the intersection of the optical axis and the image plane. $-k_{u}$ and $k_{v}$ are the vertical and horizontal scaling factors, respectively expressed in pixel/mm, assuming that the photosensitive cells of a camera are not perfectly square. Additionally, *s* is the skew parameter assuming that the two directions of the image sensors are not perfectly orthogonal. With recent devices, this parameter is very negligible in practice. The resulting camera matrix obtained is:

$$\begin{array}{ccc} K & = & \left[\begin{array}{ccc} 1021.162 & 0 & 375.077\\ 0 & 1019.759 & 244.155\\ 0 & 0 & 1 \end{array}\right] \end{array}$$

Secondly, a 3D transformation (rotation *R* and translation *t*) maps any point $\tilde{M}$ from the camera frame $R_{c}$ to a point $\tilde{Q}\left(X,Y,Z,1\right)$ in the radar frame $R_{r}$, such as:

(3) $$\tilde{M}=A\tilde{Q}$$

with *A* the extrinsic matrix parameters:

(4) $$A=\left[\begin{array}{cc} R & t\\ 0 & 1 \end{array}\right]=\left[\begin{array}{cccc} R_{11} & R_{12} & R_{13} & tx\\ R_{21} & R_{22} & R_{23} & ty\\ R_{31} & R_{32} & R_{33} & tz\\ 0 & 0 & 0 & 1 \end{array}\right]$$

Replacing $\tilde{M}$ in Equation (1) by the formula in Equation (3) provides the final transformation mapping of the 3D to 2D points as follows:

(5) $$w\tilde{p}=\left[K|0\right]A\tilde{Q}$$

## 3. Three-Dimensional Reconstruction

**Figure 2:**
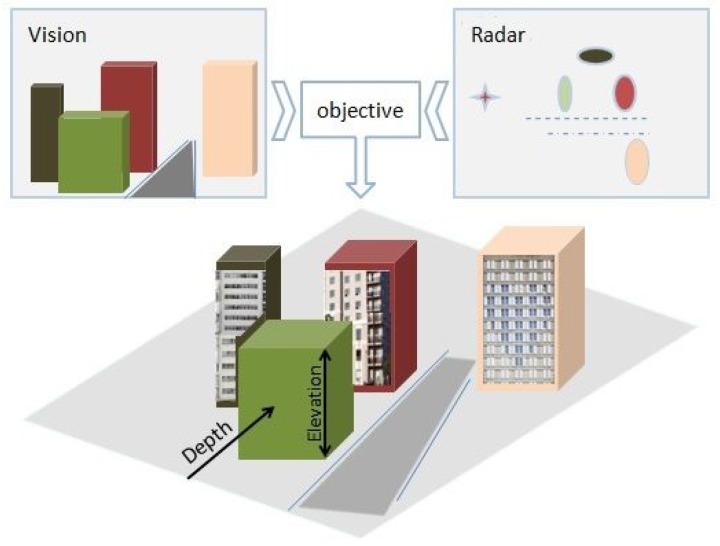
An illustration of the elevation map generation exploiting radar and vision complementarity.

Because of the geometrical projection performed by the sensors, part of the information is lost during acquisition. The 3D reconstruction of a scene is then the compensation of the missing data from two-dimensional acquisitions. The 3D reconstruction of a large-scale environment is a challenging topic. For these reasons, the proposal of a simple, robust and fast algorithm dedicated to complete such an objective represents a major interest for several applications. Our goal is to prove the concept of using radar, which is underrated for 3D reconstruction, and to build a simple 3D sensor that is easy to use by a non-expert operator, which provides textured elevation maps, as illustrated in Figure 2.

**Figure 3:**
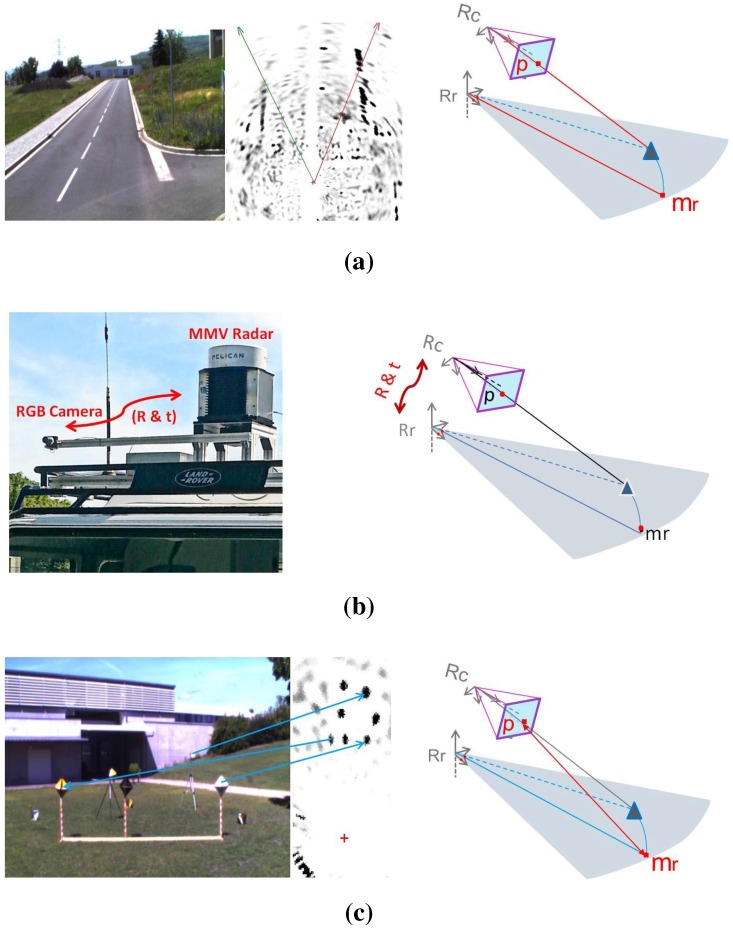
In order to achieve the 3D reconstruction, three preliminary steps must be carried out: simultaneous data acquisition by the sensors, the estimation of the transformation between the sensor frames and the extraction and matching of features from the camera image and the radar panoramic. (**a**) Data acquisition; (**b**) System calibration; (**c**) Features extraction and matching.

In order to achieve the 3D reconstruction, preliminary steps must be carried out. These steps are illustrated in Figure 3: the data acquisition should be done simultaneously by each sensor having an overlapping field of view.

The acquisitions are synchronized using GPS data. The calibration step consists of determining the transformation mapping target coordinates from one sensor frame to another.

The feature extraction and matching between the data provided by these two sensors are essential, yet difficult process, since the data are inherently different; thus, they cannot be easily compared or matched. For the current stage, further works are in progress in order to automate this step.

In order to recover the third dimension, we proceed as follows: a 3D point *Q* detected by the camera and by the radar belong, in fact, to the light ray *L* passing through the optical center of the camera, and at the same time, this belongs to a sphere *C*, centered on the radar’s antenna center, as shown in Figure 3. Therefore, its 3D coordinates are obtained by estimating the coordinate of the intersection point between the sphere *C* and the straight *L*.

The equation of the sphere is written as follows:

(6) $$\left(C\right)\;{\left(x-x_{O_{r}}\right)}^{2}+{\left(y-y_{O_{r}}\right)}^{2}+{\left(z-z_{O_{r}}\right)}^{2}=r^{2}$$

where $O_{r}\left(x_{r},y_{r},z_{r}\right)$ and *r* are the sphere center and radius, respectively, in the camera coordinate frame. Additionally, the light ray equation is Equation (1), with *w* the unknown parameter:

$$w\tilde{p}=\left[K|0\right]I_{4\times 4}\tilde{M}$$

One can write:

(7) $$\tilde{M}=\left[\begin{array}{c} K^{-1}w\tilde{p}\\ 1 \end{array}\right]=\left[\begin{array}{c} wm\\ 1 \end{array}\right]$$

and:

(8) $$m=K^{-1}\tilde{p}={\left[\begin{array}{ccc} m_{1} & m_{2} & m_{3} \end{array}\right]}^{T}$$

Our method consists of three steps: First, the scale factor *w* is to be determined. From Equation (7), *x*, *y* and *z* can be written as a function of *w*: $x=wm_{1}$, $y=wm_{2}$ and $z=wm_{3}$. Replacing *x*, *y* and *z* in Equation (6) thereby leads to a quadratic equation in *w*:

(9) $$\begin{array}{c} w^{2}\left(m_{1}^{2}+m_{2}^{2}+m_{3}^{2}\right)-2w\left(m_{1}x_{O_{r}}+m_{2}y_{O_{r}}+m_{3}z_{O_{r}}\right)\\ +(x_{O_{r}}^{2}+y_{O_{r}}^{2}+z_{O_{r}}^{2}-r^{2})=0 \end{array}$$

**Figure 4:**
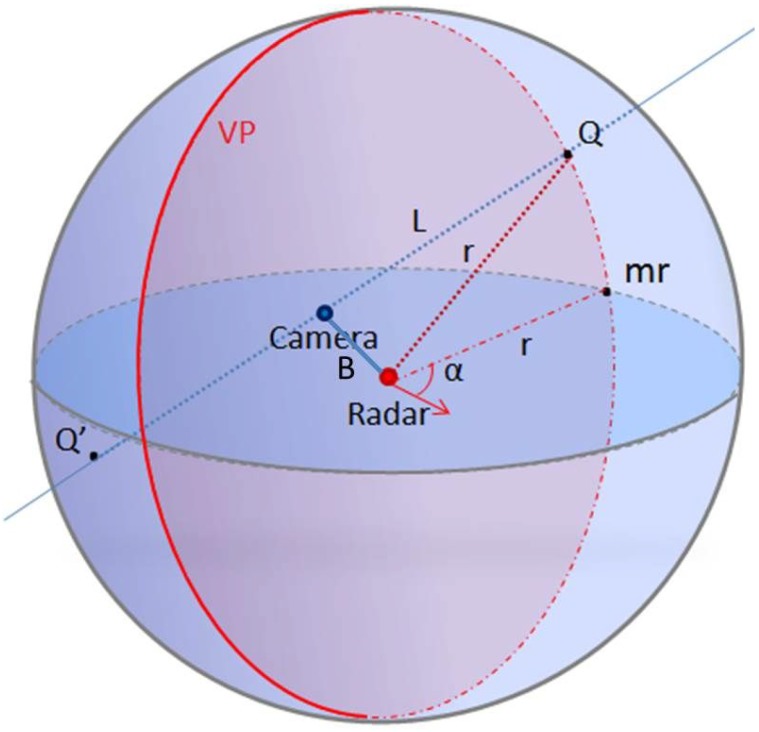
The 3D reconstructed point *Q* is the intersection of light ray *L* and the sphere *C* at *α*. $m_{r}$ is the projected 2D point on the horizontal radar plane, and VP is the vertical plane of the target at *α*.

Since we are working in a large-scale environment, the targets are usually too far compared to the baseline *B* (the distance between the radar and camera frames). Then, the camera is always inside the sphere *C*, so theoretically, two solutions for the quadratic Equation (9) exist as illustrated in Figure 4, *w* and $w^{\prime }$. From these solutions, two points $\tilde{M}{\left(x,y,z,1\right)}^{T}$ and $\tilde{M^{\prime }}{\left(x^{\prime },y^{\prime },z^{\prime },1\right)}^{T}$ relative to the vision sensor frame are deduced from Equation (7). Secondly, their coordinates in the radar frame should be computed from Equation (3):

$$\tilde{M}=A\tilde{Q}$$

One can write:

(10) $$\tilde{Q}=A^{-1}\tilde{M}\;\text{and}\;\tilde{Q^{\prime }}=A^{-1}\tilde{M^{\prime }}$$

Finally, the azimuth angles of these 3D points are computed. Thereby, the correct solution is selected by comparing the computed azimuth angles and the one measured by the radar. The calibration step is then requisite in order to determine the transformation matrix *A* and the radar center $O_{r}\left(x_{r},y_{r},z_{r}\right)$.

## 4. System Calibration

The calibration is an important factor affecting the reconstruction accuracy. Hence, it is required to develop an accurate calibration method and with a rather simple implementation.

### 4.1. Related Work

The closest work on camera-radar system calibration is the work of *S.* Sugimoto *et al.* [31]. The radar acquisitions are considered to be coplanar, since it performs a planar projection on its horizontal plane. Therefore, the transformation *A* is a homography *H* between image and radar planes, without going through the calculation of the rotation and translation matrices. For z=0 (horizontal plane), these pairs are related as shown in Equation (11):

(11) $$w\left[\begin{array}{c} u\\ v\\ 1 \end{array}\right]=H\left[\begin{array}{c} x\\ y\\ 1 \end{array}\right]$$

*H* is a 3×3 homography matrix, relating camera and radar planes.

In spite of its theoretical simplicity, this method is hard to implement. Indeed, while the canonical target is being continuously moved up and down by a mechanical system, it should be simultaneously acquired by the radar and camera. Then, pairs of matches (four pairs at least), corresponding to the exact intersection of the target with the horizontal plane of the radar, are extracted. Moreover, due to the sampling frequency, the exact positions are determined from the maximum of the intensity reflected by the target using bilinear interpolation of the measurement samples along the vertical trajectory of each target.

### 4.2. The Proposed Calibration Method

Our goal is to determine the rotation and the translation between the frames of the sensors. For an azimuth angle *α*, we have $\vec{n}=\left(\sin\left(\alpha \right),-\cos\left(\alpha \right),0\right)$, the normal to the plane VP. Since VP is a vertical plane passing by $O_{r}$, it has the following equation:

(12) $$\begin{array}{c} X\sin(\alpha )-Y\cos(\alpha )=0 \end{array}$$

The equation of the sphere *C* centered on $O_{r}\left(0,0,0\right)$ becomes:

(13) $$\left(C\right)\;{\left(X\right)}^{2}+{\left(Y\right)}^{2}+{\left(Z\right)}^{2}=r^{2}$$

From Equation (5), the 3D point $\tilde{Q}$ is expressed as a function of $\tilde{p}$, *w* and *A* as follows:

(14) $$\begin{array}{ccc} \tilde{Q} & = & A^{-1}\left[\begin{array}{c} K^{-1}w\tilde{p}\\ 1 \end{array}\right]\\ & = & \left[\begin{array}{cc} R^{T} & -R^{T}t\\ 0 & 1 \end{array}\right]\left[\begin{array}{c} wm\\ 1 \end{array}\right]\\ & = & \left[\begin{array}{cc} R^{T}wm & -R^{T}t\\ 0 & 1 \end{array}\right] \end{array}$$

From Equation (14), *X*, *Y* and *Z* can be expressed in terms of unknown *A* and *w*:

(15) $$\left\{\begin{array}{c} X=A_{11}^{-1}wm_{1}+A_{12}^{-1}wm_{2}+A_{13}^{-1}wm_{3}+A_{14}^{-1}\\ Y=A_{21}^{-1}wm_{1}+A_{22}^{-1}wm_{2}+A_{23}^{-1}wm_{3}+A_{24}^{-1}\\ Z=A_{31}^{-1}wm_{1}+A_{32}^{-1}wm_{2}+A_{33}^{-1}wm_{3}+A_{34}^{-1} \end{array}\right.$$

For *n* matches, system $\left(S_{1}\right)$ is obtained, with i=1→n and *ϵ* the residuals:

$$\left(S_{1}\right)\left\{\begin{array}{c} X_{i}^{2}+Y_{i}^{2}+Z_{i}^{2}-r_{i}^{2}=\epsilon_{1}^{i}\\ X_{i}\sin\left(\alpha_{i}\right)-Y_{i}\cos\left(\alpha_{i}\right)=\epsilon_{2}^{i} \end{array}\right.$$

The equations are expressed with respect to a parameter vector $\left[\gamma_{x},\gamma_{y},\gamma_{z},t_{x},t_{y},t_{z},w_{i}\right]$; *γ* are the three rotational angles relative to *x*, *y* and *z*. The system is underdetermined, and many solutions exist, hence the need to add more geometric constraints.

### 4.3. Inter-Distance Constraint

**Figure 5:**
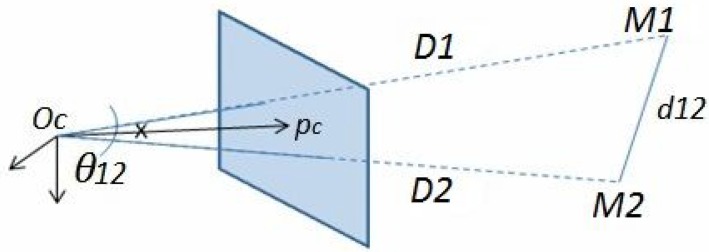
The triangle formed by M1, M2 (3D points in the camera frame) and $O_{c}$ is shown. $d_{12}$ is the Euclidean distance between M1 and M2 that is supposed to be known, and $D_{1}$, $D_{2}$ are their depths relative to $O_{c}$.

In order to calculate the scale factor *w* for each target, we propose to use the theorem of Al-Kashi [35]. This theorem is known as the “law of cosines” that generalizes the Pythagorean theorem of an unspecified triangle. The latter, applied to the triangle formed by two 3D points $M_{1}$, $M_{2}$ with $O_{c}$, as illustrated in Figure 5, gives the following equations:

(16) $$D_{1}^{2}+D_{2}^{2}-2L_{12}=d_{12}^{2}$$

where:

(17) $$L_{12}=D_{1}D_{2}\cos\left(\theta_{12}\right)$$

The only constraint for the proposed method is the *a priori* knowledge of distances between the targets used for calibration. For *n* matches with i=1→n, $D_{i}$ is the depth of the point relative to $O_{c}$, and it is related to the scale factor $w_{i}$ and the angle $\beta_{i}$ formed between the principle point $p_{c}$ and pixel $p_{i}$ by the formula:

(18) $$\begin{array}{c} D_{i}=\frac{w_{i}}{\cos(\beta_{i})} \end{array}$$

with:

(19) $$\begin{array}{c} \cos\left(\beta_{i}\right)=\frac{p_{c}^{T}{\left(KK^{T}\right)}^{-1}p_{i}}{\sqrt{\left(p_{c}^{T}{\left(KK^{T}\right)}^{-1}p_{c}\right)\left(p_{i}^{T}{\left(KK^{T}\right)}^{-1}p_{i}\right)}} \end{array}$$

$d_{ij}$ is the known distance between points, and $\theta_{ij}$ is the angle between two rays lining up the 3D points with $O_{c}$ (see Figure 5). The cosine of $\theta_{ij}$ is calculated from corresponding pixels in the image and intrinsic matrix K in this manner:

(20) $$\begin{array}{c} \cos\left(\theta_{ij}\right)=\frac{p_{i}^{T}{\left(KK^{T}\right)}^{-1}p_{j}}{\sqrt{\left(p_{i}^{T}{\left(KK^{T}\right)}^{-1}p_{i}\right)\left(p_{j}^{T}{\left(KK^{T}\right)}^{-1}p_{j}\right)}} \end{array}$$

Since we have six degrees of freedom (DOF), three for the rotation angles and three for the translation, relative to *x*, *y* and *z*, we need at least six points. With six 3D points, we have 15 inter-distances, so we obtain a system $\left(S_{2}\right)$ of 15 equations in terms of $w_{i=1\to 6}$, and $\epsilon^{i=1\to 6,j=1\to 6}$ are the residuals:

$$\left(S_{2}\right)\left\{\begin{array}{c} D_{i}^{2}+D_{j}^{2}-2L_{ij}-d_{ij}^{2}=\epsilon_{3}^{ij} \end{array}\right.$$

The system is solved by the algorithm of Levenberg–Marquardt, based on non-linear least squares optimization of the sum of squared residuals ${\left(\epsilon \right)}^{2}$, in order to determine the approximate solution as shown hereafter:

(21) $$\begin{array}{c} \sum {\left(\epsilon_{1}^{i}\right)}^{2}+{\left(\epsilon_{2}^{i}\right)}^{2}\;\text{and}\;\sum {\left(\epsilon_{3}^{ij}\right)}^{2} \end{array}$$

### 4.4. Relaxation of the Inter-Distance Constraint

**Figure 6:**
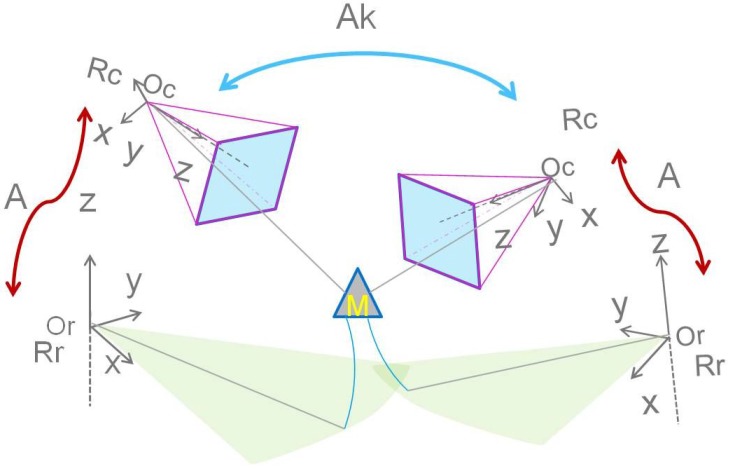
The displacement of the system around a fixed scene gives more geometric equations. An illustration of this process is shown. The matrix $A_{k}$ represent the transformation between one position and another.

For further simplification of the implementation process, we tend to relax the *a priori* inter-distance constraint, which could also be an additional source of error. In this context, geometrical equations should be added to the system in the optimization process. We propose to do so by moving the system of sensors, while keeping the captured scene fixed (see Figure 6). This allows for multiple acquisitions of the scene from different points of view and provides additional equations.

Following this change of the point of view of the acquisitions, an additional transformation matrix $A_{k}$, corresponding to a displacement *k* of the system, is to be determined. A 3D point $Q_{k_{i}}$ in the radar frame is then expressed as a function of unknown *A*, $A_{k}$ and *w* as follows:

For k=0, we have:

(22) $$Q_{k_{i}}=A^{-1}\left(w_{i}\cdot K^{-1}\cdot P_{i}\right)$$

Additionally, for k≥1, we have:

(23) $$Q_{k_{i}}=A_{k}^{-1}\cdot A_{k-1}^{-1}\cdot A^{-1}\left(w_{i}\cdot K^{-1}\cdot P_{i}\right)$$

With *i* representing the number of 3D points, and $P_{i}$ is the corresponding pixel.

Thus, $X_{k_{i}}$, $Y_{k_{i}}$ and $Z_{k_{i}}$ can be written as follows:

(24) $$\left\{\begin{array}{c} X_{k_{i}}=A_{k_{11}}^{-1}X_{k-1_{i}}+A_{k_{12}}^{-1}Y_{k-1_{i}}+A_{k_{13}}^{-1}Z_{k-1_{i}}+A_{k_{14}}^{-1}\\ Y_{k_{i}}=A_{k_{21}}^{-1}X_{k-1_{i}}+A_{k_{22}}^{-1}Y_{k-1_{i}}+A_{k_{23}}^{-1}Z_{k-1_{i}}+A_{k_{24}}^{-1}\\ Z_{k_{i}}=A_{k_{31}}^{-1}X_{k-1_{i}}+A_{k_{32}}^{-1}Y_{k-1_{i}}+A_{k_{33}}^{-1}Z_{k-1_{i}}+A_{k_{34}}^{-1} \end{array}\right.$$

Then, the following system $\left(S_{3}\right)$ of two additional equations is obtained, for each point *i* from a displacement *k*:

$$\left(S_{3}\right)\left\{\begin{array}{c} X_{k_{i}}^{2}+Y_{k_{i}}^{2}+Z_{k_{i}}^{2}-r_{k_{i}}^{2}=\epsilon_{4}^{i}\\ X_{k_{i}}\sin\left(\alpha_{k_{i}}\right)-Y_{k_{i}}\cos\left(\alpha_{k_{i}}\right)=\epsilon_{5}^{i} \end{array}\right.$$

For k=1 and i=8, we have a total of 14 unknowns and 8×2×2 equations, so a total of 32 equations; thus, the system is overdetermined. The parameter vector would be then: $\left[\gamma_{x},\gamma_{y},\gamma_{z},t_{x},t_{y},t_{z},\gamma_{x_{k}},\gamma_{y_{k}},\gamma_{z_{k}},t_{x_{k}},t_{y_{k}},t_{z_{k}},w_{i}\right]$.

The resulting cost function is then optimized using the Levenberg–Marquardt least square optimization algorithm, in order to determine the unknowns:

(25) $$\begin{array}{c} \sum {\left(\epsilon_{1}^{i}\right)}^{2}+{\left(\epsilon_{2}^{i}\right)}^{2}\;\text{and}\;\sum {\left(\epsilon_{4}^{i}\right)}^{2}+{\left(\epsilon_{5}^{i}\right)}^{2} \end{array}$$

The number of targets *i* depends on the number of displacements *k*, such that the more the system is moved, the fewer the targets that will be needed.

## 5. Uncertainty Analysis

In order to study the effect of several parameters on the accuracy of the proposed method, simulations on synthetic data were carried out using MATLAB. Sets of 3D points are randomly generated following a uniform random distribution within a cubic work space in front of the camera-radar system.

The projected pixel and the polar coordinates of each 3D point are computed using the pinhole model of the camera and the geometric model of the radar sensor, as explained in Section 2.

First, the calibration and reconstruction algorithms were tested with exact data input, and the obtained errors are around 10${}^{-6}$ on translation, 10${}^{-12}$ on rotation and around 10${}^{-14}$ for the reconstruction method. Afterwards, the simulations were extended emulating realistic cases in order to test the efficiency and the robustness of the methods with respect to several parameters, such as the number of matches, the noise level, the baseline length and the elevation of the target. Therefore, synthetic data are disrupted by uniformly-distributed random noise.

**Figure 7:**
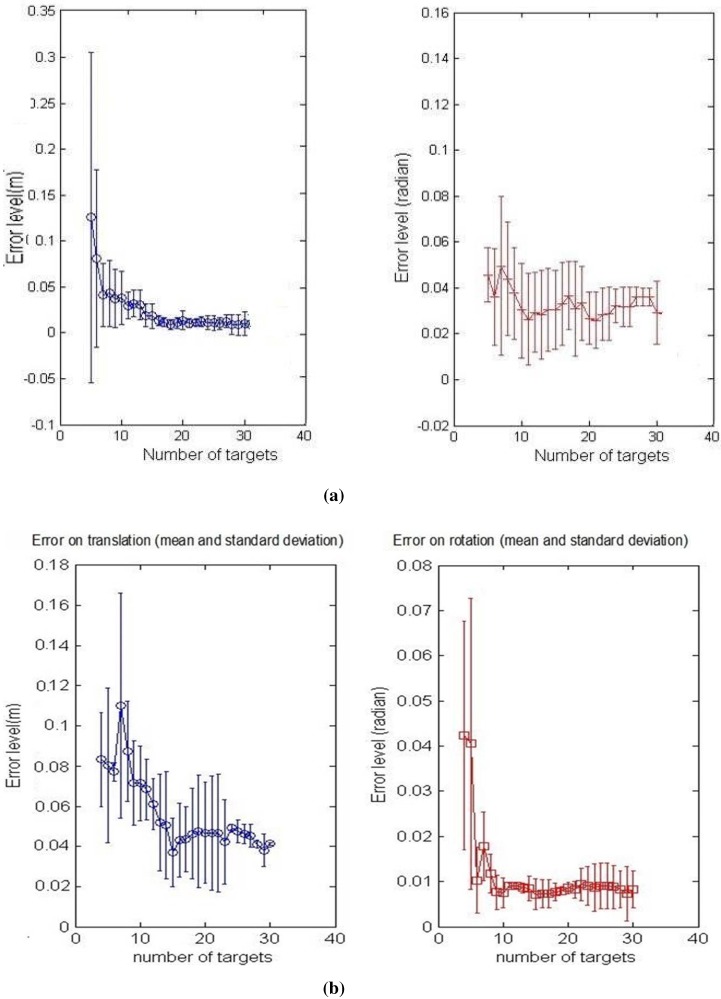
Calibration error with respect to the number of points. **Left column**: translation error in meters; **Right column**: rotation error in radians. The graphs show the mean and the standard deviation of RMSE upon six iterations. The number of matches is increased by a step of one from three to 30, and the noise level is: ±2 p, ±$2^{\circ }$ for *α*, ± 2 cm for *r*. (**a**) First calibration method; (**b**) Second calibration method.

Both calibration methods were tested, and the simulations results are discussed and compared in this section. For the first step, we added noise corresponding to ±2 pixels, ±$2^{\circ }$ for the azimuth angle and ±2 cm for the distance. The number of matched points used for the calibration process is increased by a step of onefrom five to 30 points, and the calibration, using the two constraints, respectively, is done in order to analyze the convergence of the algorithms. The mean and standard deviation of the RMS error upon six specimens are shown in Figure 7a,b.

It is noticeable that the shapes of the error curve, for both translation and rotation, are similar regardless of the method of calibration used. As might be expected, the errors decrease starting from six matches for both calibration methods, and then, they remain nearly stable. This is due to the non-linear problem that converges more precisely to the correct solution when the noisy equation system is over-determined.

It can be also noticed that the results of the second method in Figure 7b, show more accurate behavior with increasing number of points at the same noise level; this is due to a larger number of equations used for this method and, therefore, a better convergence.

**Figure 8:**
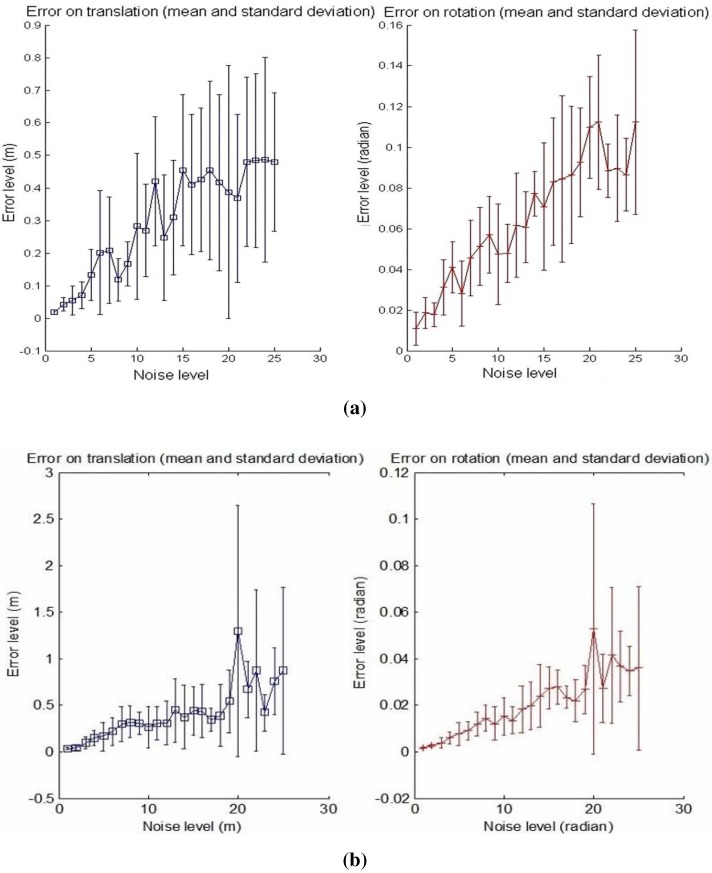
Calibration error with respect to the noise level. **Left**: translation error in meters; **Right**: rotation error in radians. The graphs show the mean and the standard deviation of RMSE upon six reiterations with 10 matches used. (**a**) First calibration method; (**b**) Second calibration method.

Secondly, in order to test the accuracy of the calibration, linearly increasing the noise level is applied to the input data starting from Level 1, corresponding to ±0.5 pixels, ±$0.5^{\circ }$ for the azimuth angle and ±5 cm for the distance, up to Level 25, corresponding to ±5 pixels, ±$0.5^{\circ }$ for the azimuth angle and ±50 cm for the distance. Error graphs are shown in Figure 8a,b.

The number of matches used for the calibration process is 10. The mean and the standard deviation of the RMS error upon six specimens are also shown for the translation and rotation results. It should be noticed that the effects of the increasing noise on the rotation and translation increase the errors: non-linear algorithms are affected by noise, and yet, our algorithms show an acceptable behavior in the presence of noisy data. The results of the second method in Figure 8b show a smaller increase compared to the first curves in Figure 8a with increasing noise level.

**Figure 9:**
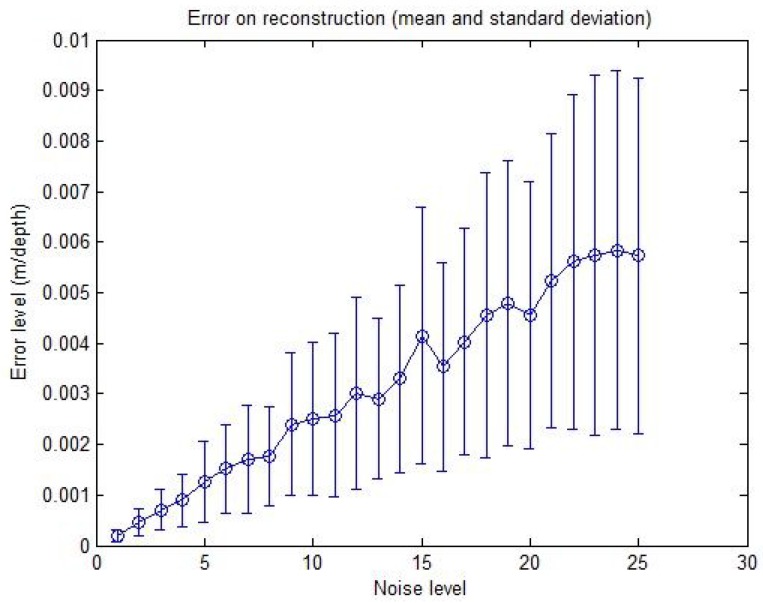
Reconstruction error with respect to the noise level. The error is in meters relative to the point depths *r*. The mean and standard deviation of the RMSE, over 50 reconstructed points, are shown.

The accuracy of the reconstruction method is similarly tested with respect to the same noise levels, and the resulting error graph is shown in the Figure 9.

The graph in Figure 9 shows the mean and standard deviation of the RMSE upon 50 reconstructed points for each level. Despite the slight increase of the error with increasing noise level, it is quite clear that the method is very robust in the presence of noise. For example, for the 25th level corresponding to ±5 pixels, ±$5^{\circ }$ for the azimuth angle and ±50 cm for the depth, the error mean is about 0.0058 m, which is a quite good result.

**Figure 10:**
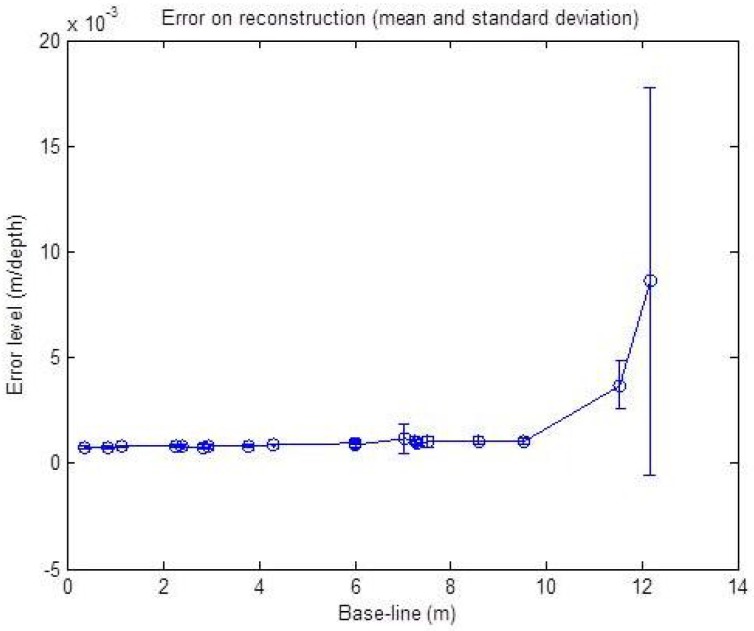
Reconstruction error with respect to the baseline with a noise level corresponding to ±2 p, ±$2^{\circ }$ for *α* and ±2 cm for *r*. The error is in meters relative to the point depths (*r*). The mean and standard deviation over 50 reconstructed points are shown.

The influence of the baseline between the sensor centers, on the reconstruction methods, is also studied with simulated noisy data. The input data are this time disrupted with a fixed noise level corresponding to ±2 pixels, ±$2^{\circ }$ and ±2 cm. The baseline width is increased from 0 cm up to 12 m in length. The resulting graph is shown in Figure 10. The graph shows the RMSE mean and standard deviation over 50 reconstructed points for each level.

**Figure 11:**
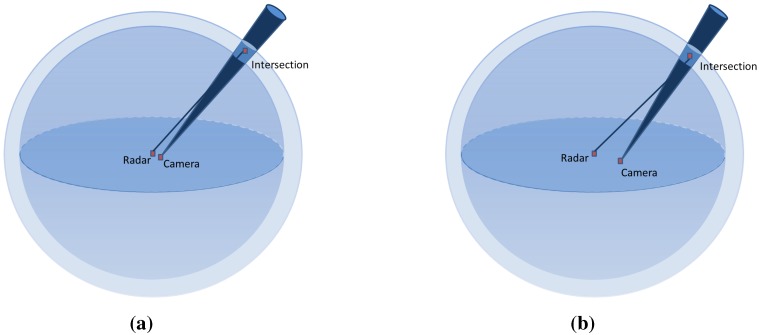
Illustration of the baseline effect on the reconstruction error. (**a**) Intersection uncertainty using a narrow baseline; (**b**) Intersection uncertainty using a wide baseline.

The result of this simulation shows a nearly stable error level for the baseline under a 10 m width. Starting from 10 m, we can see a slight increase of the error level with respect to the increasing width. This is due to the geometric constraint, as shown in Figure 11: in the presence of noise, for two different baseline widths, the intersection of the uncertainty regions of each sensor is not affected by the baseline width.

**Figure 12:**
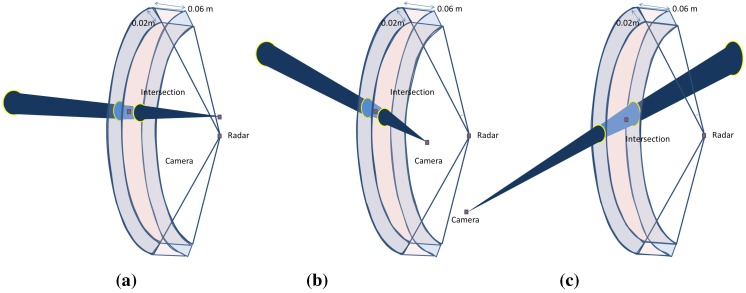
The effect of the baseline is illustrated. The intersection of the uncertainty regions of each sensor projection is also shown. (**a**) Short baseline; (**b**) Wide baseline; (**c**) Wide baseline.

However, a very slight increase is observed when the baseline width is greater than the depth of the target, as shown in Figure 12. However, this is not considered an issue for large-scale scenes and can be ignored, since the camera is often closer to the radar than the surrounding targets.

**Figure 13:**
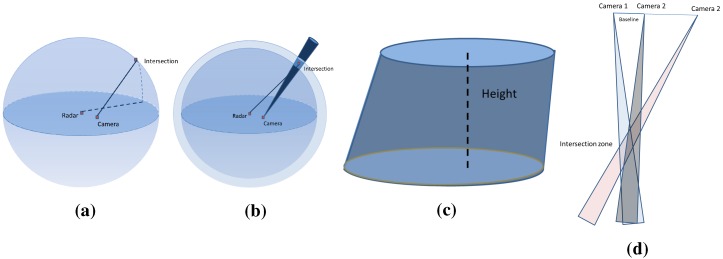
The intersection of the uncertainty regions of each sensor’s projection: (**a**) the ideal case of the geometric reconstruction; (**b**) introducing uncertainty regions of each sensor to the geometric model; (**c**) the error intersection region; (**d**) uncertainity intersection region in respect to the baseline for the stereo reconstruction method.

According to this study on the baseline parameter, we can consider from the above results that our reconstruction method does not require a constrained baseline width, unlike the vision-based methods, where the baseline width has a strong especial affect on the results for large-scale scenes. Indeed, in vision-based approaches, distant targets require a longer baseline in order to reduce the range uncertainty of the 3D reconstruction, as illustrated in Figure 13d. Only having further afield points of view leads to a decreased common area between the two acquisitions, thereby affecting the complexity of the image registrations. The error on the pixel in the image camera is derived from the camera projection error and the extraction uncertainty of the targets. The error on the radar data is the uncertainty for the azimuth angle of $1^{\circ }$ , corresponding to 0.06, and for the depth of 0.02 m. In the ideal case, the reconstruction is done by calculating the intersection between a straight and a sphere, as illustrated in the Figure 13a. However, introducing the uncertainty of each sensor to the geometric model, the reconstruction error is then the intersection of the cone corresponding to the camera uncertainty zone and the inter-sphere region corresponding to the radar uncertainty zone (see Figure 13b). The intersection zone between the sphere and the cone can be approximated to an ellipse. Therefore, the error corresponds to a truncatedoblique cone, as illustrated in Figure 13c, having a volume $v=\pi /3(ab_{baseellipse}+\left(ab_{baseellipse}\right)\left(a^{\prime }b_{topellipse}^{\prime }\right)+a^{\prime }b_{topellipse}^{\prime })height$, where a,b and $a^{\prime },b^{\prime }$ correspond to the major and minor axes of the base ellipse and top ellipse, respectively. The height of the truncatedcone is equal to the difference between the maximum and minimum depth in the uncertainty zone.

**Figure 14:**
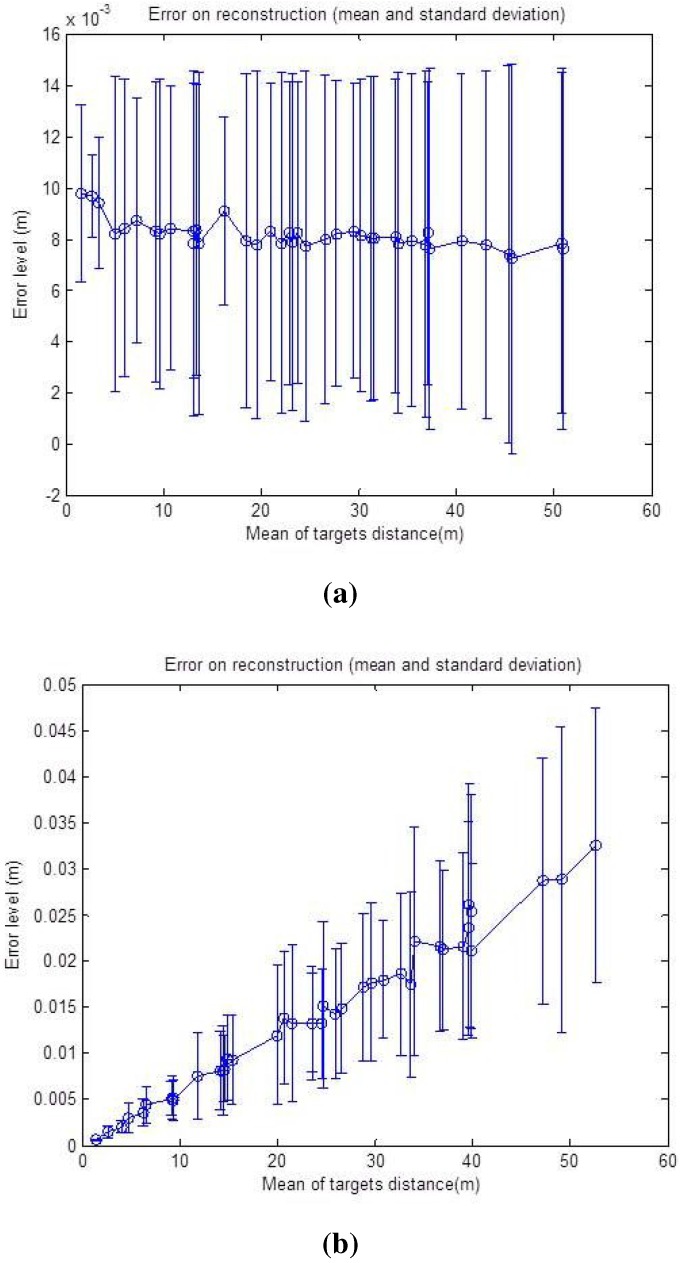
Reconstruction error with respect to the mean depth of the targets. The error is in meters. The mean and standard deviation of the RMSE, over 50 reconstructed points, are shown. (**a**) Noise added corresponding to 0 p, ±$2^{\circ }$ for *α* and ±2cm for *r*; (**b**) Noise added corresponding to ±2 p, $0^{\circ }$ for *α* and 0 cm for *r*.

**Figure 15:**
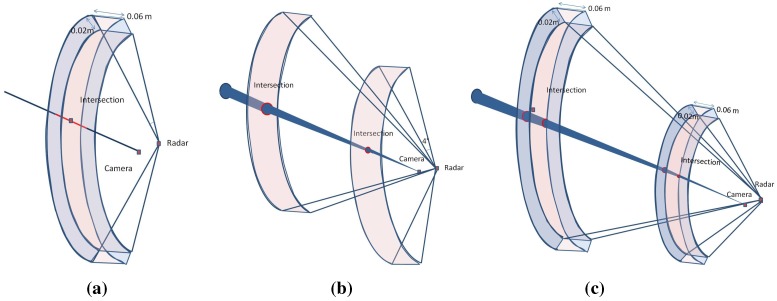
Illustration of the baseline effect on the reconstruction error. (**a**) Noise added only to radar data; (**b**) Noise added only to camera data; (**c**) Noise added to both camera and radar data.

The same noise level is added on simulated data in order to study the effects of targets depths on the error level for the reconstruction algorithm. The resulting graphs are shown in Figure 14 and Figure 15, respectively.

First, the noise is added just to the radar data (zero for the pixel, ±$2^{\circ }$ for *α* and ±2 cm for *r*) and then to the data from the camera (±2 for the pixel, $0^{\circ }$ for *α* and 0 cm for *r*), as the uncertainty zone changes differently for the two sensors with the increasing distance of the targets. One can remark from the graph in Figure 14a that the reconstruction error is oscillating around 1 cm, and it is almost stable compared to a growing target depth. This is explained by the fact that the precision of the radar measurement is stable with respect to the distance. The geometric interpretation is illustrated in Figure 15a, where the error of the reconstruction is only depending on the error of the depth of the target, which is independent of distance. The azimuth provided by the radar is not taken into account in the reconstruction Equation (9). On the other hand, the graph in Figure 14b shows that the uncertainty zone of the camera increases with distance and, thus, increases the reconstruction error, as can be seen in Figure 15b.

**Figure 16:**
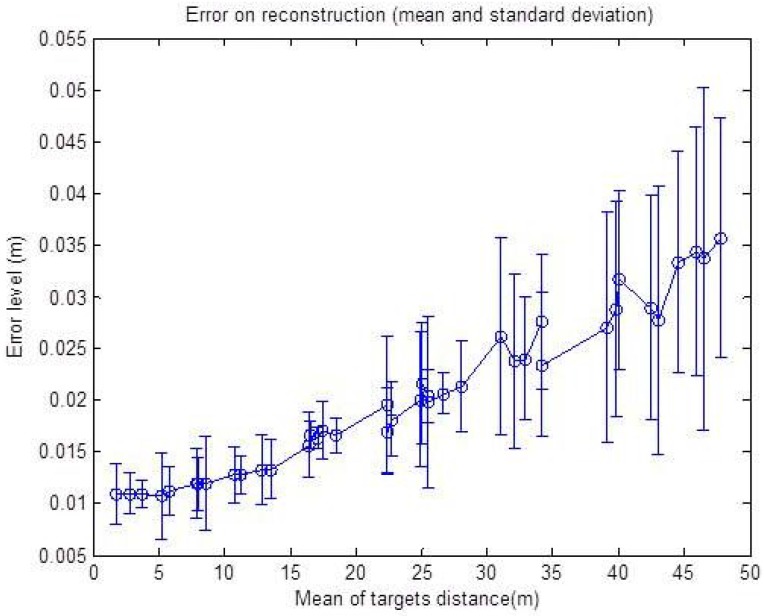
Reconstruction error with respect to the mean depth of the targets with a noise level corresponding to ±2 p, ±$2^{\circ }$ for *α* and ±2 cm for *r*. The error is in meters. The mean and standard deviation of the RMSE, over 50 reconstructed points, are shown.

Thereby, the effect of this parameter on the reconstruction method is shown in Figure 16. As we can see, the graph shows a rising error level caused by the camera rising uncertainty zone, but the minimum error level at the beginning of the curve is this time affected by the noise on the depth *r*. This is illustrated in Figure 16; for a shallow depth, the uncertainty zone of the camera is smaller than that of the radar, and then, it increases with distance.

## 6. Experimental Results

**Figure 17:**
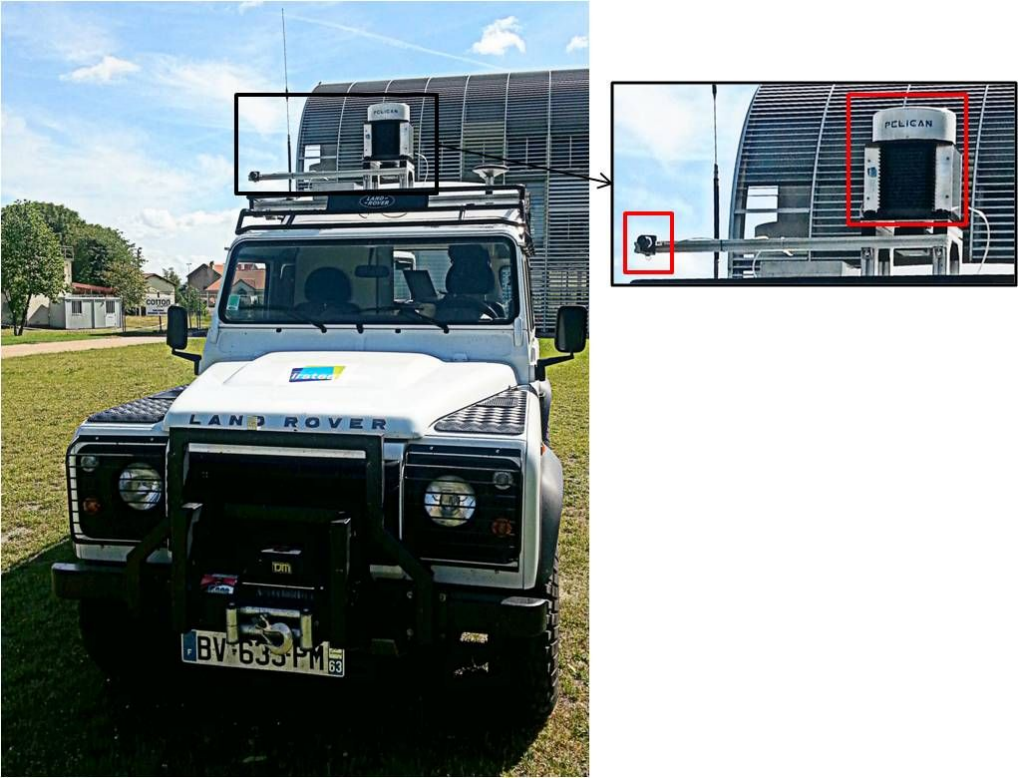
Radar and camera system. The image to the right presents the zoom in of the sensors system (the radar to the right and the camera to the left).

In order to validate the theory of the proposed methods, experiments on real data were carried out. The radar and the camera were mounted in a fixed configuration on the top of a vehicle, in front of the scene (for the current stage, the radar antenna rotates $360^{\circ }$, but the camera is stable). The radar is called K2Pi and has been developed by Irstea Institute. The optic sensor used is uEye by IDS (Imaging Development Systems). The camera and radar characteristics are listed in Table 1. A GPS mounted on the vehicle has been used for the synchronization of the data acquisition carried out by these two sensors. The system is shown in Figure 17. The baseline between the sensors is about 60 cm.

**Table 1:** Camera and radar characteristics.

Camera Characteristics	
Sensor technology	CMOS
Sensor size	4.512×2.880 mm
Pixel size	0.006 mm
Resolution in pixel (h×v)	752×480
Focal distance	8 mm
Viewing angle	43× $25^{\circ }$
**Radar Characteristics**	
Carrier frequency	24 GHz
Antenna gain	20 dB
Range	3 to 100 m
Angular resolution	$4^{\circ }$
Distance resolution	1 m
Distance precision	0.02 m
Viewing angle	360× $20^{\circ }$

**Figure 18:**
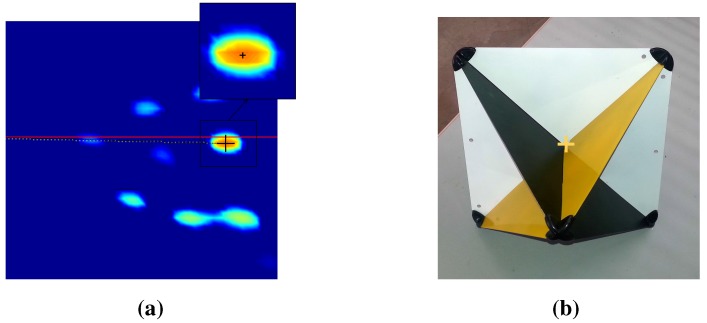
Center detection of targets in both the camera image and radar panoramic. (**a**) Radar target extraction; (**b**) Camera target extraction.

**Figure 19:**
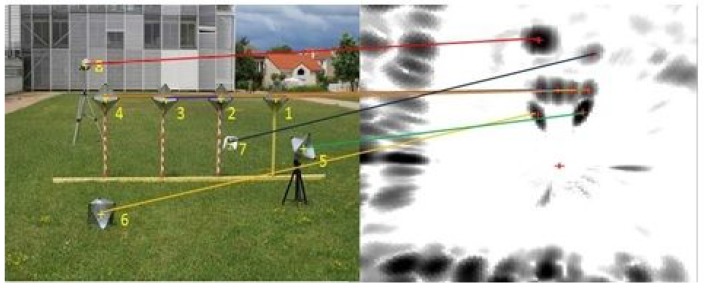
An image and a panoramic of targets. The targets are numbered from 1 to 8: one Luneburg lens and seven trihedral corners. The yellow crosses indicate the centers of the targets. The variations of amplitude of the reflected signal are introduced by the nature and orientation of each target. Manually-extracted matches between the image and the PPI are shown.

At the beginning, the system should be calibrated; we placed eight canonical targets in front of the sensor system. Metallic canonical targets (Figure 18) are highly reflective regardless of their position relative to the radar and with a small cross-section. The depth of the targets is chosen to be slightly close: between 6 m and 14 m for the first calibration method and between 10 m and 17 m for the second calibration method. The sensor system is placed in a manner that will enable clear acquisitions of the targets by the camera and the radar simultaneously (thus, there is a limited depth), and it should also be compatible with the context of the practical usage of the system. For the second calibration method, the system was moved several times in order to capture the scene from different points of view. This is in order to facilitate feature extraction from the images. The features extracted for the calibration experiments are the target centers. Centers are matched manually, and Figure 19 shows the corresponding pixels and radar targets extracted in the image of the camera and the panoramic image.

The extraction of target centers in the radar images is done as follows:

First, the target centers are selected manually; then, Gaussian filtering is performed to be more specific. Gaussian estimation corresponding to the target is done, and the maximum position is then detected, as seen in Figure 18a. Finally, we calculate the polar coordinates of the target. The center extraction method of the targets from the images is illustrated in Figure 18b: the targets are painted in order to contrast the faces of the targets, so that their centers can be readily detected thanks to their special geometrical shape, using the Harris corner detector [36]. Thus, the accuracy is assumed to be sub-pixel, as in classical camera calibration processes.

**Figure 20:**
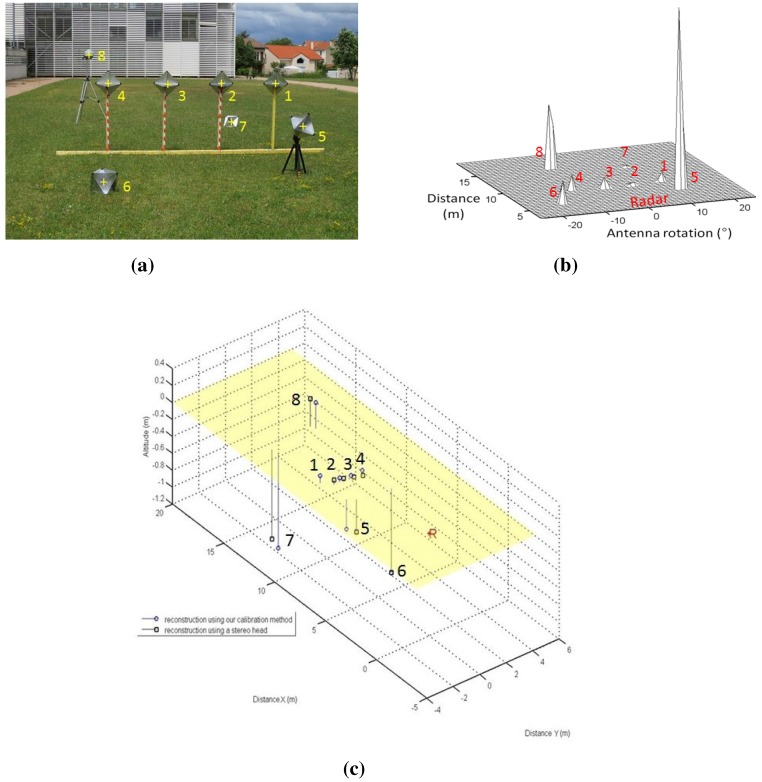
Reconstruction results using the inter-distance constraint for calibration. (**a**) Camera image of the eight canonical targets: one Luneburg lens and seven trihedral corners. The yellow crosses indicate the center of the targets; (**b**) Radar image with eight canonical targets; (**c**) The reconstruction results of both of our reconstruction methods (circular points) and the stereo head method as the ground truth (squared points). The radar position is indicated by the letter R.

In order to assess both results of the calibration method and to validate the reconstruction method, the eight targets were placed at different heights and depths. The reconstruction is obtained in the radar frame. Figure 20 and Table 2 represent the results of the reconstruction technique, using the method of Section 4.3, and reconstruction results using a stereo head, used as the ground truth. The inter-distances between the targets centers are measured, and an image and panoramic of the eight targets in a random configuration are captured. The resulting 3D point clouds were registered using the ICP (iterative closest point) algorithm. The RMSE of the reconstruction method is about 0.63 m with a standard deviation of 0.15 m. The results show a realistic error for the 3D reconstruction of targets at a mean depth of 12 m.

**Table 2:** Reconstruction results in m using the first calibration method.

	Target Coordinates in Meters with the Stereo Head (Ground Truth)							
X	5.41	7.45	7.47	7.44	7.53	4.59	12.53	13.01
Y	1.32	1.45	0.70	−0.03	−0.78	−0.68	2.24	−1.03
Z	−0.38	0.05	0.02	−0.01	−0.06	−0.92	−1.05	0.33
	**Targets Coordinates in Meters with the Developed Method**							
X	5.50	7.55	7.53	7.46	7.51	4.57	12.64	12.98
Y	0.83	0.86	0.11	−0.62	−1.37	−1.12	1.40	−1.89
Z	−0.35	0.05	0.01	−0.03	−0.08	−0.89	−1.15	0.21
	**Error in Meters (Euclidean Distance)**							
	0.49	0.60	0.59	0.59	0.59	0.44	0.85	0.87
	Error mean in meters = 0.63							
	Error standard deviation in meters = 0.15							

**Table 3:** Reconstruction results in m using the second calibration method.

	Targets Coordinates in Meters with the Stereo Head (Ground Truth)							
X	14.21	14.40	14.58	16.85	17.91	18.55	20.04	21.70
Y	−3.08	−0.02	2.05	−4.53	0.53	3.88	−3.21	−0.15
Z	−0.19	−0.18	−0.17	−1.10	0.28	−0.90	0.98	−0.61
	**Targets Coordinates in Meters with the Developed Method**							
X	12.57	12.56	12.53	15.20	15.94	16.59	18,56	19,52
Y	−3.09	0.03	2.09	−4.59	0.55	3.96	−3,20	−0,058
Z	0.09	0.14	0.19	−1.03	0.35	−0.85	0,88	−0,79
	**Error in Meters (Euclidean Distance)**							
	0.05	0.04	0.06	0.05	0.05	0.03	0.08	0.11
	Error- mean in meters = 0.058							
	Error- standard deviation in meters = 0.024							

**Figure 21:**
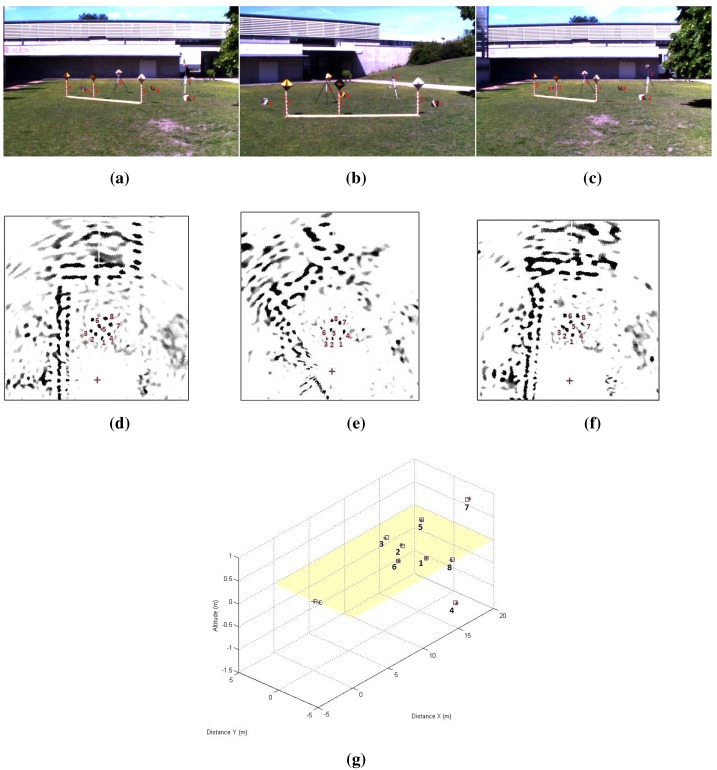
Top line: Camera images of the eight canonical targets. middle line: Radar images with eight canonical targets. The reconstruction results from both our reconstruction methods (circular points) and the stereo head method as the ground truth (squared points). The radar position is indicated by the letter R. (**a**) First position; (**b**) Second position; (**c**) Third position; (**d**) First position; (**e**) Second position; (**f**) Third position; (**g**) Reconstruction results.

For the current stage, the system is not moved. Figure 21 and Table 3 represent the reconstruction results using the second calibration method. The results are also registered and compared to the ground truth stereo head reconstruction of the same scene. The RMSE mean is about 0.058 m with a standard deviation of 0.024 m on X,Y and *Z*.

**Figure 22:**
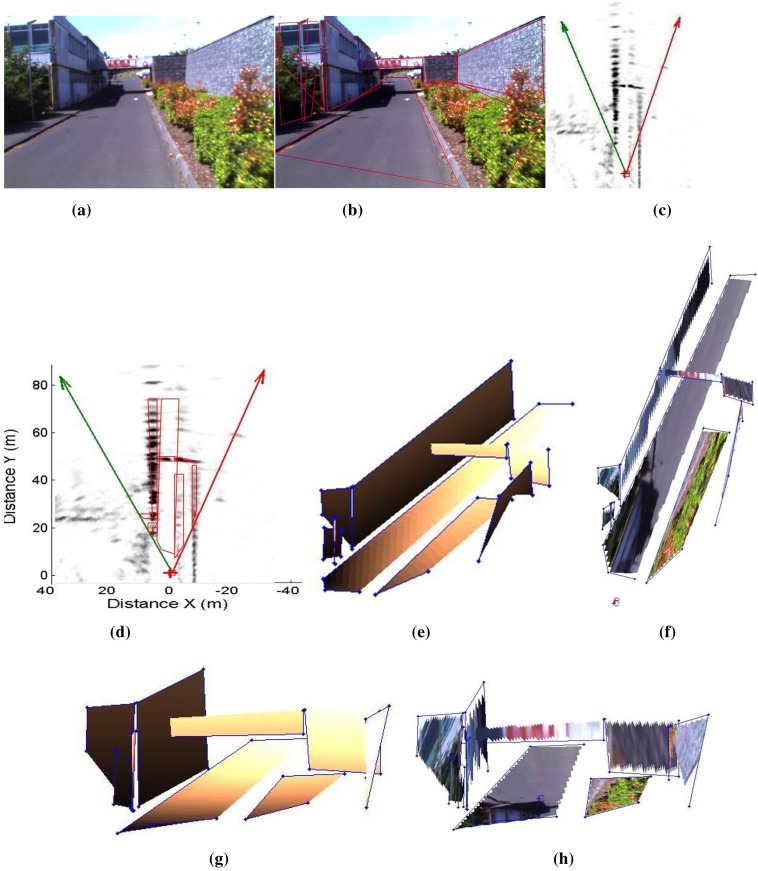
Results of a reconstructed urban scene using the camera/radar system and the second calibration method. The results are enhanced with texture mapping (this figure is clearer in color). (**a**) Camera image of an urban scene; (**b**) Segmented image (polygons are shown in red); (**c**) Part of the radar image of the same scene; (**d**) Segmented radar image; (**e**) Results of the reconstruction using Delaunay triangulation; (**f**) Enhanced results with texture; (**g**) Another view of the 3D results; (**h**) Another view of the 3D results.

**Figure 23:**
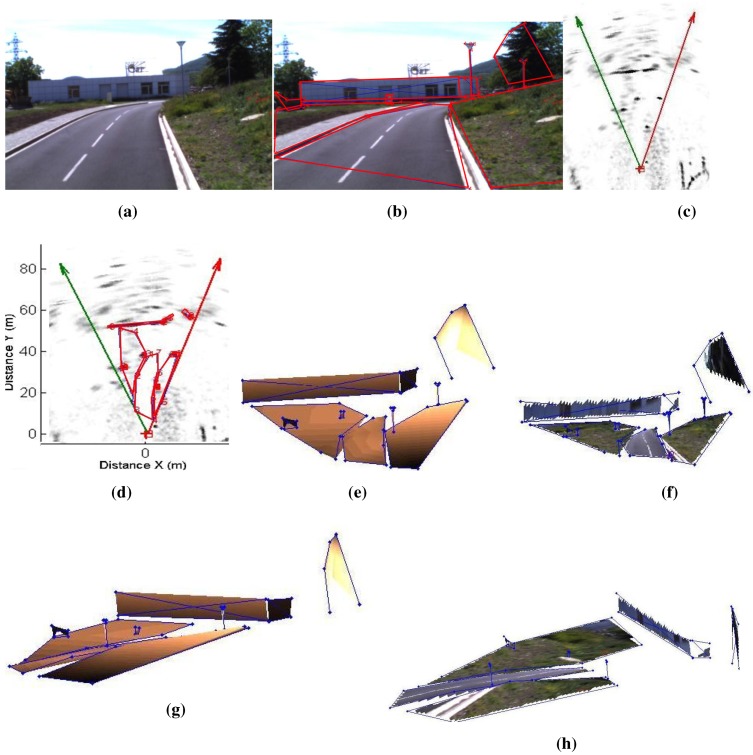
Results of a reconstructed urban scene using the camera/radar system, and the second calibration method. The results are enhanced with texture mapping (this figure is clearer in color). (**a**) Camera image of the an urban scene; (**b**) Segmented camera image; (**c**) Part of the radar image of the same scene; (**d**) Segmented radar image; (**e**) Results of the reconstruction using Delaunay triangulation; (**f**) Enhanced results with texture; (**g**) Another view of the 3D results; (**h**) Another view of the 3D results.

Figure 22 and Figure 23 show the results of reconstructed urban scenes using our system. The camera/radar system is calibrated; the second calibration method is used for this experiment. Eight targets are captured by the system from three different points of view, as shown in Figure 21. These results are interpreted qualitatively since no numerical ground truth results are available. The segmentation and matching of the data provided by the sensors are done interactively. Polygons are extracted from the images covering the targets of interest, and then their vertices are matched by pairs. This step is to be automated in future work. The matched points are then reconstructed and the polygons are plotted using the Delaunay triangulation algorithm, as shown in Figure 22e and Figure 23e. Finally, texture mapping is done in order to enhance the representation of the reconstructed map. In fact, the interest of this sensor fusion is shown in the example in Figure 22, as the radar provides no information about the elevation of the bridge, this later being detected as a barrier. The elevation and vertical occupation of the bridge are extracted from the image of the camera. Therefore, this ambiguity is eliminated after the reconstruction process.

## 7. Conclusions

In this paper, we presented a geometrical algorithm for 3D reconstruction of large-scale scenes using an MMW radar and a camera. To our knowledge, this type of data fusion has not been used for large-scale outdoor reconstruction. In contrast to other reconstruction methods, the proposed method is easy to implement and uses very few input data. Afterward, we addressed the spatial calibration problem. It can be concluded from the state of the art that this step is usually hard to carry out for this type of sensor fusion. We described a simple method of calibration of the system, using two different constraints: the inter-distance and the pose constraint. From these constraints, additional equations are derived, and then, the system of non-linear equations is solved by a non-linear optimization algorithm. The simulated and experimental results prove the feasibility of our methods and quit a good performance in the presence of noise. The accuracy of these methods with respect to several parameters (the number of targets, the noise level and the baseline width) has been studied. Finally, we presented the experimental validation with real data and a qualitative validation of urban scene reconstruction.

For the current stage, features are extracted and matched manually; therefore, RANSAC-like algorithms must be set up in order to make automatic matching. The intended matching technique will consist of first segmenting both radar and camera images into regions using standard techniques and then applying a region matching algorithm, which will use both geometrical and observation model criteria. On the other hand, camera rotation is an interesting step, since the radar has a panoramic angle of view, which allows for large coverage of the area. Further real-time reconstruction experiments of urban and semi-urban scenes should be carried out with an added texture to enhance the resulting map.

## References

[1] Henry P., Krainin M., Herbst E., Ren X., Fox D. (2012). RGB-D mapping: Using Kinect-style depth cameras for dense 3D modeling of indoor environments. Int. J. Robot. Res..

[2] Pfitzner C., Antal W., Hess P., May S., Merkl C., Koch P., Koch R., Wagner M. 3D Multi-Sensor Data Fusion for Object Localization in Industrial Applications. Proceedings of the 41st International Symposium on Robotics, ISR/Robotik 2014.

[3] Guivant J.E., Marden S., Pereida K. Distributed Multi Sensor Data Fusion for Autonomous 3D Mapping. Proceedings of the IEEE 2012 International Conference onthe Indoor Positioning and Indoor Navigation (IPIN).

[4] Bhagawati D. Photogrammetry and 3-D Reconstruction-the State of the Art. Proceedings of the ASPRS 2000.

[5] Kordelas G., Perez-Moneo Agapito J., Vegas Hernandez J., Daras P. State-of-the-Art Algorithms for Complete 3D Model Reconstruction. Proceedings of the Engage Summer School.

[6] Musialski P., Wonka P., Aliaga D.G., Wimmer M., Gool L., Purgathofer W. (2013). A Survey of Urban Reconstruction. Comput. Graph. Forum.

[7] Gallup D., Frahm J.M., Mordohai P., Yang Q., Pollefeys M. Real-Time Plane-Sweeping Stereo with Multiple Sweeping Directions. Proceedings of the 2007 IEEE Conference on Computer Vision and Pattern Recognition.

[8] Pollefeys M., Nistér D., Frahm J.M., Akbarzadeh A., Mordohai P., Clipp B., Engels C., Gallup D., Kim S.J., Merrell P. (2008). Detailed Real-Time Urban 3d Reconstruction from Video. Int. J. Comput. Vis..

[9] Royer E., Lhuillier M., Dhome M., Lavest J.M. (2007). Monocular Vision for Mobile Robot Localization and Autonomous Navigation. Int. J. Comput. Vis..

[10] Kim C., Kim B., Kim H. (2013). 4D CAD Model Updating Using Image Processing-Based Construction Progress Monitoring. Autom. Constr..

[11] Aydin C.C. (2014). Designing Building Façades for the Urban Rebuilt Environment with Integration of Digital Close-Range Photogrammetry and Geographical Information Systems. Autom. Constr..

[12] Yang M.D., Chao C.F., Huang K.S., Lu L.Y., Chen Y.P. (2013). Image-Based 3D Scene Reconstruction and Exploration in Augmented Reality. Autom. Constr..

[13] Furukawa Y., Ponce J. (2010). Accurate, Dense, and Robust Multiview Stereopsis. IEEE Trans. Pattern Anal. Mach. Intell..

[14] Zhu Z., Kanade T. (2008). Modeling and Representations of Large-scale 3D Scenes. Int. J. Comput. Vis..

[15] Lafarge F., Keriven R., Brédif M., Vu H.H. (2013). A Hybrid Multiview Stereo Algorithm for Modeling Urban Scenes. IEEE Trans. Pattern Anal. Mach. Intell..

[16] Zhang Y., Li Q., Lu H., Liu X., Huang X., Song C., Huang S., Huang J. (2015). Optimized 3D Street Scene Reconstruction from Driving Recorder Images. Remote Sens..

[17] Rouveure R., Monod M., Faure P. High Resolution Mapping of the Environment with a Ground-Based Radar Imager. Proceedings of the IEEE International Radar Conference-Surveillance for a Safer World.

[18] Smisek J., Jancosek M., Pajdla T. (2013). 3D with Kinect. Consumer Depth Cameras for Computer Vision.

[19] Schindhelm C.K. Evaluating SLAM Approaches for Microsoft Kinect. Proceedings of the Eighth International Conference on Wireless and Mobile Communications.

[20] Forlani G., Nardinocchi C., Scaioni M., Zingaretti P. (2006). Complete Classification of Raw LiDAR Data and 3D Reconstruction of Buildings. Pattern Anal. Appl..

[21] Stamos I., Allen P.K. (2000). 3-D Model Construction Using Range and Image Data. Proc. IEEE Comput. Vis. Pattern Recogn..

[22] Bok Y., Choi D.G., Kweon I.S. (2014). Sensor Fusion of Cameras and a Laser for City-Scale 3D Reconstruction. Sensors.

[23] Cheng L., Tong L., Chen Y., Zhang W., Shan J., Liu Y., Li M. (2013). Integration of LiDAR Data and Optical Multi-View Images for 3D Reconstruction of Building Roofs. Opt. Lasers Eng..

[24] Duraisamy P., Jackson S., Namuduri K., Alam M.S., Buckles B. (2013). Robust 3D Reconstruction Using LiDAR and N-Visual Image. Proc. SPIE.

[25] Williams K., Olsen M.J., Roe G.V., Glennie C. (2013). Synthesis of Transportation Applications of Mobile LiDAR. Remote Sens..

[26] Bertozzi M., Bombini L., Cerri P., Medici P., Antonello P.C., Miglietta M. Obstacle Detection and Classification Fusing Radar and Vision. Proceeding of the IEEE Intelligent Vehicles Symposium.

[27] Roy A., Gale N., Hong L. Fusion of Doppler Radar and Video Information for Automated Traffic Surveillance. Proceedings of the 12th International Conference on IEEE Information Fusion.

[28] Hofmann U., Rieder A., Dickmanns E.D. (2003). Radar and Vision Data Fusion for Hybrid Adaptive Cruise Control on Highways. Mach. Vis. Appl..

[29] Wang T., Zheng N., Xin J., Ma Z. (2011). Integrating Millimeter Wave Radar with a Monocular Vision Sensor for on-Road Obstacle Detection Applications. Sensors.

[30] Bombini L., Cerri P., Medici P., Alessandretti G. (2006). Radar-Vision Fusion for Vehicle Detection. Int. Workshop Intell. Transp..

[31] Sugimoto S., Tateda H., Takahashi H., Okutomi M. (2004). Obstacle Detection Using Millimeter-Wave Radar and Its Visualization on Image Sequence. Int. Conf. Pattern Recogni..

[32] El-Natour G., Ait-Aider O., Raphael R., Francois B., Faure P. (2015). Sensor Fusion of Cameras and a Laser for City-Scale 3D Reconstruction. Int. Conf. Comput. Vis. Theory Appl..

[33] Skolnik M.I. (2001). Introduction to Radar Systems.

[34] Bouguet J.Y. Camera Calibration Toolbox for Matlab. http://www.vision.caltech.edu/bouguetj/calib_doc/.

[35] Kennedy E.S. (1952). A Fifteenth-Century Planetary Computer: al-Kāshī’s Tabaq al-Manāteq. II. Longitudes, Distances, and Equations of the Planets. Isis.

[36] Harris C., Stephens M. (1988). A Combined Corner and Edge Detector. Alv. Vis. Conf..

